# Association between Neuropsychiatric Symptoms and Cognitive Functions in Subjects with Probable Alzheimer's Disease in Kinshasa.

**DOI:** 10.1002/alz70859_101984

**Published:** 2025-12-25

**Authors:** Guy Gikelekele

**Affiliations:** ^1^ CNPP/University of Kinshasa, Kinshasa, Kinshasa Congo (The Democratic Republic of the)

## Abstract

**Background:**

Many studies in Western countries have examined the association between neuropsychiatric symptoms (NPS) and cognitive functions in dementia, primarily focusing on Alzheimer’s disease (AD). However, few studies have evaluated this association in Sub‐Saharan African countries. This study examines the association between NPS and cognitive functions in older adults with probable AD in Kinshasa, Congo (DRC).

**Method:**

A total of 1432 participants were recruited by convenience sampling in Kinshasa, DRC. They underwent the CSID and the AQ. Out of these, 114 participants were retained, including 56 considered to have probable AD and 58 considered to have normal cognition. The two groups were matched by sex, age, and education level. The Neuropsychiatric Inventory (NPI) was used to determine psychiatric symptoms, and the African Neuropsychology Battery (ANB) for cognitive functions. Descriptive statistics, including means, standard deviations, and frequencies, were conducted. The association between NPS and cognitive function was established using multiple linear regression.

**Result:**

The most frequent NPS were irritability (56%), anxiety (46%), sleep disturbance (44%), depression (42%), appetite disorders (35%), aberrant motor behavior (21%), and agitation (19%). A statistically significant association was found between anxiety and the proverb test (95% CI, p=0.03), and between sleep disturbance and visuospatial memory (95% CI, p=0.05). A trend of association was observed between anxiety and visuospatial memory (95% CI, p=0.06).

**Conclusion:**

Some NPS are part of the manifestations of AD and are associated with specific cognitive functions. This highlights the importance of using the NPI and ANB in the diagnosis of probable AD.

